# Associating Liver Partition and Portal Vein Ligation for Staged Hepatectomy (ALPPS) in Children with Advanced Hepatoblastoma—Lessons from a Case Series and Literature Review

**DOI:** 10.3390/children13070957

**Published:** 2026-07-20

**Authors:** Hanna Garnier, Maciej Murawski, Ewelina Wojciechowska, Oleksandr Kalinchuk, Katarzyna Sinacka, Ewa Izycka-Swieszewska, Piotr Czauderna

**Affiliations:** 1 Department of Pediatric Surgery and Urology for Children and Adolescents, Medical University of Gdansk, Smoluchowskiego 17, 80-210 Gdansk, Polandewelina.wojciechowska@gumed.edu.pl (E.W.);; 2 Surgical Department at St. Nicholas Hospital of the First Medical Association, Pylypa Orlyka Street 4, 79059 Lviv, Ukraine; 3 Department of Radiology, Medical University of Gdansk, Smoluchowskiego 17, 80-210 Gdansk, Poland; 4 Department of Pathology and Neuropathology, Medical University of Gdansk, Smoluchowskiego 17, 80-210 Gdansk, Poland

**Keywords:** advanced hepatoblastoma, liver, pediatric surgery

## Abstract

Background: Liver transplantation is the standard treatment for children with advanced hepato-blastoma when complete resection is not feasible. However, transplantation may be contraindicated because of persistent metastatic disease, severe comorbidities, poor clinical condition, or donor-related limitations. Associating Liver Partition and Portal Vein Ligation for Staged Hepatectomy (ALPPS) has emerged as a potential rescue strategy in highly selected patients. This study evaluated the feasibility, safety, and oncological outcomes of ALPPS in pediatric hepatoblastoma. Methods: A retrospective analysis was performed of four consecutive children with advanced hepatoblastoma who underwent classical ALPPS between 2013 and 2025 in two specialized centers. Patient characteristics, indications, future liver remnant (FLR) volumetry, perioperative outcomes, complications, and oncological follow-up were reviewed. Results: The median age at surgery was 22 months. In all patients, the FLR was considered insufficient for one-stage hepatectomy, leading to ALPPS. Rapid hypertrophy of the FLR was achieved in every case, allowing completion of the second stage after a median of 8.5 days (range, 7–11 days). FLR volume increased by 50–89% following the first stage. Despite successful liver hypertrophy, outcomes remained poor in three patients. One patient died intraoperatively from venous air embolism during the second stage, two died from disease recurrence despite aggressive multimodal treatment, and one remains in complete clinical and radiological remission following ALPPS and adjuvant chemotherapy. Conclusions: ALPPS reliably induces rapid FLR hypertrophy and may provide a potentially curative option for carefully selected children when liver transplantation is not feasible. However, its substantial perioperative risk and generally unfavorable oncological outcomes support its role only as a rescue procedure in experienced pediatric hepatobiliary centers. Further multicenter studies are needed to better define indications and patient selection.

## 1. Introduction

Liver transplantation is the most commonly chosen option for advanced hepatoblastoma. However, there is a group of patients for whom organ transplantation is not a viable or appropriate option. Transplantation carries significant consequences and represents a substantial burden on the patient, being associated with the need for lifelong immunosuppression, which in turn predisposes to numerous potential long-term complications, including an increased risk of infections, impaired growth and development, post-transplant lymphoproliferative disorder, renal toxicity, biliary and metabolic complications, de novo malignancies, nephrotoxicity from calcineurin inhibitors, cardiovascular complications, as well as graft rejection [1,2,3].

In pediatric hepatoblastoma, the most clinically relevant contraindication to liver transplantation is the presence of persistent extrahepatic disease, particularly unresectable pulmonary metastases or other metastatic disease that cannot be rendered disease-free prior to or at the time of transplantation. Additionally, in some cases, the patient’s overall condition—such as severe comorbidities, poor performance status, or uncontrolled infection—may preclude eligibility for transplantation. Furthermore, practical barriers including the unavailability of a suitable donor, prolonged waiting times, and the patient’s low body weight may further limit the feasibility of transplantation. In such cases, a concept introduced in pediatric surgical oncology for marginally resectable liver tumors, Associating Liver Partition and Portal Vein Ligation for Staged Hepatectomy (ALPPS), may be the only surgical treatment option available.

Therefore, in all patients included in the present series, liver transplantation was carefully evaluated but ultimately considered either contraindicated or not feasible before ALPPS was proposed as a salvage surgical option.

**Figure 1:**
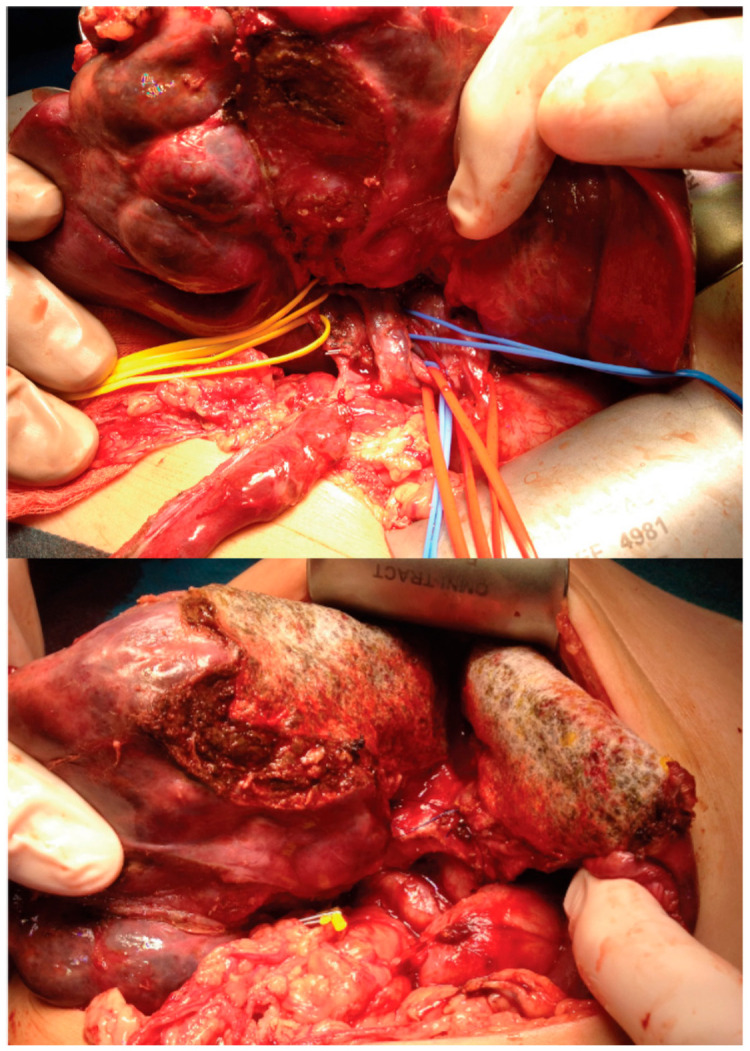
First stage of ALPPS; division of the liver parenchyma and ligation of the portal vein supplying the portion of the liver planned resection.

**Figure 2:**
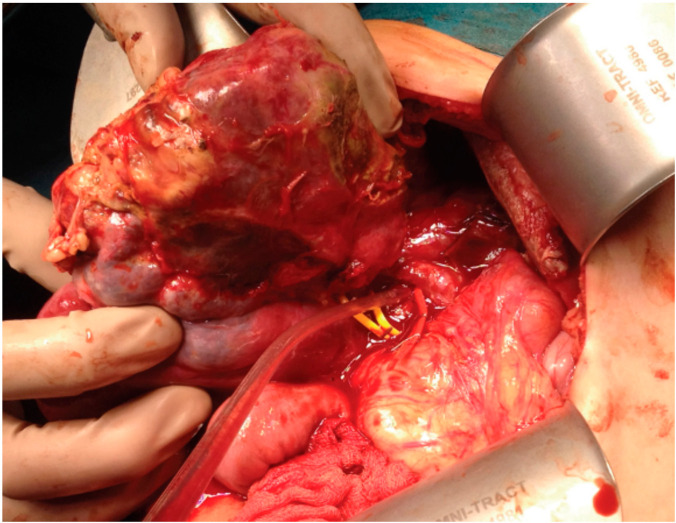
Second stage of ALPPS (7 days after): removal of the right lobe of the liver with the tumor.

The original ALPPS procedure, introduced in 2007 and first formally described in 2011 by Baumgart et al. [4], is a technique that allows for the removal of an extensive part of the liver in two stages. In the first stage of the procedure, the liver parenchyma is divided according to a preplanned line, ensuring that the tumor is entirely contained within the liver segment that will be removed. Subsequently, the branch of the portal vein supplying the planned resected portion of the liver is ligated. At this stage, the operation ends without removing the liver segment along with the tumor, Figure 1. By utilizing the liver’s remarkable regenerative capacity, the healthy portion of the liver that is spared has the potential to grow to a size capable of assuming the function of the entire liver within 7–14 days. A future liver remnant (FLR) of 20% (30% for chemotherapy-damaged livers) is recommended as an absolute minimum to maintain normal liver function, whereas 40% for people with underlying cirrhosis or hepatitis [5,6]. In the second surgery (7–14 days after the first stage), the liver segment containing the tumor is removed, Figure 2.

Over the years, various modifications of the classical ALPPS method have been described. Akhaladze et al. proposed performing the first stage of the procedure laparoscopically in pediatric patients [7]. On the other hand, there are reports in the literature regarding the use of sequential portal vein embolization (PVE) and radiofrequency ablation (RFA) (PVE + RFA) during the first stage of the procedure, which leads to a lower risk of complications and eliminates the need for two laparotomies [8,9]. However, most reports concern adult patients, for whom the procedure is performed for different diagnoses than in children [10,11]. Despite promising results in the adult patient group, the application of this method remains controversial in the pediatric patient group.

In the present study, we report a retrospective series of pediatric patients with hepatoblastoma who underwent classical ALPPS between 2013 and 2025 in two pediatric hepatobiliary centers, one in Poland and one in Ukraine. The aim of this study was to evaluate the feasibility, safety, and oncological outcomes of ALPPS in children with advanced hepatoblastoma and to discuss its role as a rescue surgical strategy in highly selected patients for whom liver transplantation was not feasible.

## 2. Materials and Methods

This retrospective case series included pediatric patients with hepatoblastoma who underwent ALPPS in two pediatric hepatobiliary centers. Patient eligibility for ALPPS was determined following multidisciplinary discussion involving pediatric surgeons, pediatric oncologists, radiologists, and transplant specialists. ALPPS was considered only in patients for whom conventional liver resection was deemed unsafe because of an insufficient future liver remnant (FLR) and liver transplantation was contraindicated or not feasible because of oncological, clinical, or logistical reasons. Preoperative volumetric assessment of the FLR was performed using contrast-enhanced CT or MRI. In our centers, ALPPS was considered when the estimated FLR was ≤25% of the total liver volume (TLV).

### 2.1. First Case Presentation

The 3-year-old patient, with a complex medical history including extreme prematurity, hydrocephalus, psychomotor delay, sensorineural deafness, and a history of necrotizing enterocolitis, presented with a 9.4 × 9.0 × 9.3 cm right hepatic lobe mass invading the portal vein. The mass was classified as PRETEXT II P+. Preoperative imaging (CT/MRI) revealed an exophytic lesion involving liver segments 5 and 6, with documented tumor thrombus within the right branch of the portal vein. Notably, portal venous cavernous transformation was identified, attributed to neonatal umbilical vessel catheterization. The patient’s baseline assessment also noted a congenitally small left hepatic lobe. The patient underwent neoadjuvant chemotherapy, which resulted in a significant reduction in tumor size (2.8 × 2.7 × 1.2 cm) and resolution of the portal vein thrombus. Following this, the tumor was reclassified as POSTTEXT II P−. A bisegmentectomy (segments 5 and 6) was performed. Histopathological evaluation confirmed R1 resection status, identifying neoplastic cells within the vasculature in close proximity (<1 mm) to the resection margin. Postoperatively, alpha-fetoprotein (AFP) levels trended upward despite the absence of radiographically detectable disease. Given the clinical progression, the patient’s comorbidities—which precluded liver transplantation—and the inadequate volume of the future liver remnant (FLR), the decision was made to perform an Associating Liver Partition and Portal Vein Ligation for Staged Hepatectomy (ALPPS) rather than a single-stage right hemihepatectomy. Formal volumetric assessment confirmed that the FLR volume was insufficient for a single-stage extended right hemihepatectomy, with a baseline volume of 35.8 cm^3^ (<11% of the total liver volume). Following the first stage of ALPPS, interval imaging demonstrated significant hypertrophy of the FLR to 60.5 cm^3^, representing a 69% increase in functional liver volume, which allowed for the completion of the procedure. Pre-ALPPS imaging confirmed that segment 4 was disease-free, though it visually corroborated the limited volume of the left lobe. The patient underwent the ALPPS procedure. Histopathological analysis again revealed focal neoplastic emboli and equivocal resection margins, underscoring the aggressive biological behavior of the malignancy. Despite aggressive surgical management, serum AFP levels continued to rise without clear imaging evidence of macroscopic recurrence. In an effort to address the biochemical evidence of disease, the patient received chimeric antigen receptor T-cell (CAR-T) therapy. This intervention yielded a transient remission; however, the disease eventually recurred in liver segment 4. The recurrence presented as a new lesion, distinct from the prior resection margins, as evidenced by serial cross-sectional imaging comparisons with the disease-free pre-ALPPS scans. Final surgical resection of segment 4 was performed. Unfortunately, the biochemical trend of rising AFP levels persisted, signaling ongoing recurrence, which ultimately led to the patient’s demise 44 months following the initial diagnosis.

### 2.2. Second Case Presentation

A 3-year-old girl was diagnosed with hepatoblastoma, epithelial–mesenchymal type, PRETEXT 4, with metastasis to the bones and lungs. Her initial alpha-fetoprotein (AFP) level was 1,950,131 IU/mL. The patient received chemotherapy according to the SIOPEL 4 HR protocol. Initial MRI revealed a tumor measuring 15 × 18 × 16 cm (4320 cm^3^). Following preoperative chemotherapy, the tumor size decreased to 7.6 × 9 × 8 cm (547.2 cm^3^), and the tumor was reclassified as POSTTEXT 3 (V-, P-, E-, R-, M+, disease-free segments 6/7 and 2/3). Due to persistent lung metastases after preoperative chemotherapy, the patient was disqualified from liver transplantation, although the tumor size had decreased to 7.6 × 9 × 8 cm (547.2 cm^3^), making ALPPS the only potentially curative surgical option. During the first stage of ALPPS, the right branch of the portal vein was ligated, and the liver parenchyma was divided between segments 2, 3, and 4, as well as in the upper part of the liver in the fissure between the right and middle hepatic veins. The portal triad supplying segment 4 was also ligated. Nine days later, it was confirmed that the future liver remnant (FLR) had significantly hypertrophied, increasing from 100.62 cm^3^ to 152.88 cm^3^, representing a 50% growth. In the second stage of ALPPS, the right hepatic artery was ligated, and the right lobe was separated from the inferior vena cava and the caudate lobe. Shortly after the tumor resection, at the very last stage of hepatectomy, a significant drop in blood pressure was observed, followed by cardiac arrest. Intensive resuscitation efforts were initiated, and an air embolism in the right ventricle was diagnosed, confirmed by intraoperative echocardiography. Despite attempts to aspirate air through a central venous catheter placed in the right ventricle and manually filling the ventricle with transfused blood, as well as filling the surgical field with saline and occluding the inferior vena cava (IVC), return of spontaneous circulation could not be achieved. After more than an hour of unsuccessful resuscitation, intraoperative death of the patient was confirmed. Histopathological findings included hepatoblastoma of the epithelial–mesenchymal type. The lymph nodes near the hepatoduodenal ligament and retroperitoneal lymph nodes were free of tumor. The resection margin was free of tumor, and the surgery was macroscopically and microscopically radical.

### 2.3. Third Case Presentation

A 3-month-old male infant, born prematurely at 30 weeks with a birth weight of 950 g, was diagnosed with a centrally located hepatoblastoma involving liver segments 7 and 8. His complex medical history included suspected corpus callosum hypoplasia, a history of CMV infection, and clinical suspicion of West syndrome. The patient was initially diagnosed with a PRETEXT II hepatoblastoma localized to segments 7 and 8. Imaging revealed the tumor was in close proximity (<1 cm) to the confluence of the right and middle hepatic veins, the portal vein bifurcation, and the right portal vein branch, while the intrahepatic IVC was located approximately 1 cm from the lesion. Segments 4a, 1 (caudate lobe), and 3 were confirmed to be free of disease. The patient initiated the treatment protocol in Ukraine; neoadjuvant SIOPEL 3 chemotherapy (four cycles of cisplatin) was performed, which resulted in a decrease in AFP levels from 39,903 IU/mL to 5533 IU/mL and a modest reduction in tumor size from 5 × 3.5 × 3.9 cm to 4.3 × 3.1 × 3 cm. However, due to the outbreak of war, the patient was evacuated, resulting in a significant delay in the therapeutic regimen. Following the patient’s transfer to a specialized center outside the conflict zone, the tumor was reclassified as POSTTEXT III without any annotation factors (caudate lobe and segment 3 free of lesions). Because the patient’s prematurity-related comorbidities and low body weight rendered a standard extended right hepatectomy unsafe, and given the inadequate volume of the future liver remnant (FLR), an ALPPS procedure was chosen. Formal preoperative volumetry confirmed the FLR (segments 2/3) was insufficient to support a single-stage resection, and mesohepatectomy was deemed unfeasible due to the tumor’s proximity to the middle hepatic vein and the high-take-off branch to segment 4b. Primary liver transplantation was not a viable option due to the patient’s poor clinical status and logistical constraints. During the first stage of ALPPS, intraoperative findings identified tumor involvement in segments 8 and 4a extending toward the falciform ligament, which was more extensive than pre-operative imaging had suggested. The first stage was performed successfully, and seven days later, the second stage was completed, confirming an FLR hypertrophy from 27.5 cm^3^ to 48.12 cm^3^ (a 75% increase in volume), which allowed for the completion of the procedure. The right lobe and segment 4b were resected. Histopathological analysis confirmed mixed epithelial and mesenchymal hepatoblastoma with a 3 mm minimal parenchymal margin; however, tumor tissue was present at the transection line of the middle hepatic vein. On postoperative day 7, a 67 × 42 × 73 mm biloma in the resection bed was identified and drained. During this procedure, intraoperative ultrasound revealed early recurrence with three 4–6 mm nodules in the caudate lobe and a 3 mm lesion in segment 3, likely representing occult multifocal disease or microscopic vascular seeding, as these segments had appeared free of lesions on pre-ALPPS imaging. Despite subsequent rescue chemotherapy (SIOPEL 4, block B), the disease progressed, and the patient was disqualified from salvage transplantation due to disseminated disease, ultimately passing away 6 months after the ALPPS procedure.

### 2.4. Fourth Case Presentation

A 6.5-month-old infant presented with a massive hepatic tumor, with AFP levels exceeding 30,000 IU/mL and imaging consistent with embryonal hepatoblastoma (PRETEXT IV, V3 + P2 + E + M-). Following neoadjuvant chemotherapy (SIOPEL 4), the patient demonstrated significant tumor regression, with the lesion shrinking from 11.1 × 10 × 8.7 cm to 5.7 × 4.2 × 3.2 cm, reclassified as POSTTEXT III (V2/P1/E0/F0/R0/M0). Residual disease involved segments 1, 4, and 8, with definitive inferior vena cava (IVC) invasion and critical proximity to the left hepatic vein (<1 mm).

Surgical management presented significant challenges: liver transplantation was precluded by parental disqualification (blood group incompatibility and chronic hepatitis B), the lack of a suitable cadaveric donor, and the patient’s low body weight (8 kg), which carries a prohibitively high risk of graft arterial thrombosis. Furthermore, chemotherapy-induced hepatotoxicity—evidenced by recurrent transaminase elevations (>8× ULN)—raised concerns regarding the functional capacity of the residual liver parenchyma. Consequently, a two-stage hepatectomy using the ALPPS (Associating Liver Partition and Portal vein ligation for Staged hepatectomy) technique was selected. The primary objectives were to ensure sufficient functional liver remnant (FLR) and to leverage compensatory hypertrophy to increase the anatomical distance between the tumor and the left hepatic vein, thereby facilitating an R0 resection.

The pre-Stage 1 FLR (segments 2–3) was 409 cm^3^. Following the first stage, rapid hypertrophy occurred, with the FLV increasing to 776 cm^3^ (89% gain) within 7 days. Eight days after the first stage, a right trisectionectomy with IVC reconstruction was performed. The intraoperative findings confirmed that the induced hypertrophy successfully shifted the vascular anatomy, increasing the margin between the tumor and the left hepatic vein to approximately 1 cm. Seven days post-resection, the liver volume reached 2100 cm^3^ (+413% relative to pre-Stage 1 baseline). The patient completed adjuvant SIOPEL IV chemotherapy and remains in complete clinical and radiological remission 17 months post-surgery. This case demonstrates that ALPPS is a viable and effective alternative to transplantation in infants with locally advanced hepatoblastoma, particularly when vascular proximity and underlying parenchymal vulnerability complicate conventional single-stage resection.

## 3. Results

Between 2013 and 2025, four ALPPS procedures were performed. The median age at the time of surgery was 22 months. All patients had a diagnosis of hepatoblastoma. In each case, the future liver remnant (FLR) after right hepatectomy with tumor removal was deemed insufficient to permit a one-stage resection. Notably, all patients demonstrated rapid hypertrophy of the FLR, with the second stage performed within 7–11 days (median: 8.5 days), Table 1. The first patient initially underwent a bisegmentectomy. Histopathological evaluation revealed the presence of microvascular neoplastic emboli at the resection margins, and following the completion of adjuvant chemotherapy, the patient exhibited a progressive rise in alpha-fetoprotein (AFP) levels. Due to these findings, the case was subjected to extensive international multidisciplinary consultations. The patient was primarily considered for liver transplantation; however, they were disqualified due to poor general condition. Consequently, it was decided that a right hemihepatectomy was necessary to achieve oncological radicality. This approach was initially deemed technically unfeasible, as the left liver lobe was too small to support adequate hepatic function post-resection. To overcome this, an ALPPS procedure was performed. The perioperative course was uneventful, and sufficient hypertrophy of the left lobe was successfully achieved. Despite these efforts, the patient died three years after the procedure due to local disease recurrence. The second patient died intraoperatively during the second stage due to venous air embolism at the final resection step. The third patient died six months postoperatively due to rapid disease recurrence. The fourth patient has remained in remission for 17 months following the ALPPS procedure.

## 4. Discussion

ALPPS, a relatively new treatment option for patients with marginally resectable liver tumors, remains a technically challenging and high-risk procedure in pediatric liver oncology. Its first reported use in a pediatric patient—a 6-year-old child with hepatoblastoma—was published by Chan et al. in 2014 [12]. The first series of pediatric patients undergoing ALPPS was reported by Wiederkehr et al. in 2015 [13]. The fundamental principle of ALPPS is that it allows for rapid hypertrophy of the future liver remnant (FLR), thereby helping to prevent postoperative hepatic insufficiency [14]. However, it must be emphasized that ALPPS is not a standard of care; rather, it is a complex surgical strategy that should be approached with extreme caution. In advanced cases of hepatoblastoma, liver transplantation remains the first choice and the gold standard of treatment. Based on our experience and the currently available evidence, ALPPS cannot currently be considered a standard treatment option for pediatric hepatoblastoma but rather a highly selective rescue strategy in carefully selected patients. Long-term oncological outcomes for ALPPS remain uncertain, and the procedure carries a substantial risk of complications, with reported morbidity rates ranging from 16% to 64%, particularly in early adult series and select pediatric reports [15].

The debate over whether extreme liver resection—including ALPPS—or liver transplantation represents the superior therapeutic approach remains ongoing. Liver transplantation offers excellent survival rates, with reported 5-year overall survival reaching approximately 80–85%. In contrast, the drawbacks of ALPPS include: (1) the potential to promote tumor growth; (2) the fact that rapid hypertrophy does not always translate into adequate liver function; (3) the technical difficulty of partitioning the liver, which can alter the surgical field and complicate the identification of resection margins; and (4) the overall unfavorable long-term oncological outcomes observed in most reported cases.

Beyond the technical feasibility of ALPPS, our case series highlights several factors that may influence oncological outcome. Two of the four patients experienced either early recurrence or death despite successful completion of the procedure and adequate hypertrophy of the future liver remnant. This suggests that technical success alone does not necessarily translate into favorable oncological outcomes. In our series, adverse features included aggressive tumor biology, vascular invasion, microscopic residual disease, persistently elevated or rising AFP levels despite treatment, and metastatic disease. These observations suggest that tumor biology and disease burden may be more important determinants of long-term outcome than the ability to achieve complete technical resection. Our experience also emphasizes the importance of careful patient selection. The only patient with sustained disease-free survival had favorable tumor response to neoadjuvant chemotherapy, complete macroscopic resection, and no evidence of disseminated disease. Conversely, patients with persistent metastatic disease, vascular involvement, or unfavorable tumor biology experienced poor oncological outcomes despite technically successful ALPPS. These findings support the concept that ALPPS should be reserved for highly selected patients after multidisciplinary evaluation.

To date, data on the use of ALPPS in pediatric patients remain scarce. The only comprehensive systematic review and individual patient meta-analysis, published by Fuchs et al. in 2022, summarized all pediatric ALPPS and PVE cases reported in the literature up to that time and included 21 patients [16]. Since its publication, only a small number of additional pediatric case reports have become available, reflecting the continued rarity of this procedure. An overview of the currently available pediatric literature is presented in Table 2. Given the limited evidence (consisting primarily of case reports and small case series) and the high-risk nature of the procedure, qualification for ALPPS must be strictly individualized. It should be reserved exclusively for patients with otherwise unresectable tumors where transplantation is not a viable option, and it must be performed only in specialized centers with multidisciplinary pediatric hepatobiliary expertise [17].

While recent publications from 2023 to 2025 highlight a growing interest in ALPPS for centrally located hepatoblastomas or large benign tumors, it remains a controversial approach [18,19]. These reports further support the technical feasibility of ALPPS but do not substantially expand the evidence regarding optimal FLR thresholds or long-term oncological outcomes because of the very limited number of patients. Modifications such as replacing the first stage with portal vein embolization (PVE) have been described as a potentially safer, minimally invasive alternative to achieve FLR hypertrophy [9]. However, there is currently no universally accepted minimal FLR threshold in pediatric patients. In adult liver surgery, an FLR of approximately 20% is generally considered sufficient for healthy livers, 30% for chemotherapy-injured livers, and 40% for cirrhotic livers. In contrast, pediatric practice relies largely on institutional experience and individualized assessment. Owing to the higher liver-volume-to-body-weight ratio and greater regenerative capacity in children, several authors have suggested that FLR values of approximately 15–20% may be sufficient in selected pediatric patients with otherwise healthy liver parenchyma. Nevertheless, robust evidence is lacking, and careful multidisciplinary evaluation remains essential before considering ALPPS.

The present study has several limitations. It is a retrospective case series including only four patients treated over a prolonged period in two institutions. Therefore, our observations should be interpreted cautiously and should not be considered sufficient to establish definitive recommendations regarding the role of ALPPS in pediatric hepatoblastoma.

In conclusion, based on our limited institutional experience and the currently available literature, ALPPS should be regarded as a highly selective rescue strategy rather than a standard treatment option for pediatric hepatoblastoma. It may be considered only in carefully selected patients in whom liver transplantation is contraindicated or not feasible and should be performed exclusively in experienced multidisciplinary hepatobiliary centers. Although rapid hypertrophy of the future liver remnant can be reliably achieved, favorable technical results do not necessarily translate into favorable oncological outcomes. As the pediatric liver-volume-to-body-weight ratio is higher than in adults, an FLR of <20% may sometimes be sufficient, potentially reducing the need for such aggressive surgical interventions in selected patients [20,21,22]. For benign tumors, or in exceptional malignant cases where complete resection is mandatory but transplantation is not an option, ALPPS remains a potential alternative. However, given the rarity of the procedure, its considerable morbidity, and the limited evidence currently available, larger prospective multicenter studies with longer follow-up are needed to better define patient selection criteria, safety, and long-term oncological outcomes before broader recommendations can be made.

**Table 2:** Published pediatric cases of ALPPS reported in the literature.

Study	Year, Country	No of ALPPS Patients	No of ALPPS Patients	Diagnosis	Main Findings
Fuchs et al. [16]	2022, Germany	Systematic Review	21	Various	Comprehensive review of pediatric experience
Garcia Moreno et al. [17]	2024, Colombia	Case Series	3	HBL	Centrally located tumors
Sretenovic et al. [18]	2025, Serbia	Case Report	1	Rhabdoid Tumor	First ALPPS for Rhabdoid Tumor
Caballes et al. [22]	2025, Philippines	Case Report	1	Mesenchymal hamartoma	ALPPS for mesenchymal hamartoma

## Figures and Tables

**Table 1:** Comparative characteristics of patients undergoing ALPPS procedure.

	Patient 1	Patient 2	Patient 3	Patient 4
Diagnosis	HBL	HBL	HBL	HBL
Age at procedure (months)	25	37	6	10
Liver growth (%)	68.9	50	275	89
Interval between stages (days)	11	9	7	7

## Data Availability

The data presented in this study are available on request from the corresponding author. The data are not publicly available due to privacy and ethical reasons.
